# Supplementary material for: Multi-GeV electron-positron beam generation from laser-electron scattering
Source: Sci Rep. 2018 Mar 16;8:4702. doi: 10.1038/s41598-018-23126-7 (PMC5856856; doi:10.1038/s41598-018-23126-7)
Supplement: Supplementary file 1 — Supplementary Information [file 41598_2018_23126_MOESM1_ESM.pdf]

Supplemental material for "Multi-GeV electron-positron beam generation from laser-electron scattering"

Marija Vranic[1,2,*], Ondrej Klimo[2,3], Georg Korn[2], and Stefan Weber[2]

[1]GoLP/Instituto de Plasmas e Fusão Nuclear, Instituto Superior Técnico, Universidade de Lisboa, 1049-001 Lisbon, Portugal
[2]Institute of Physics of the CAS, ELI-Beamlines Project, Na Slovance 2, Prague, 182 21, Czech Republic
[3]Faculty of Nuclear Sciences and Physical Engineering, Czech Technical University in Prague, Brehova 7, Prague, 115 19, Czech Republic
*marija.vranic@ist.utl.pt

A general solution for particle motion in a plane wave with arbitrary initial conditions

This section sumarises the analytical solution for relativistic particle motion in a plane electromagnetic wave with an arbitrary initial phase presented in Ref.[1]. Particle energy lost due to the classical radiation reaction is included in the model.

The wave amplitude is taken to be constant, and there is no feedback on the wave itself. According to Ref[1], relativistic 4-velocity within a linearly polarized wave can be expressed as:

$$u^\mu(\phi) = \frac{1}{h(\phi)}\left\{u_0^\mu + \frac{1}{\rho_0}[h^2(\phi)-1]n^\mu - \frac{1}{\rho_0}I_1(\phi)\frac{ef_1^{\mu\nu}}{m}u_{0,\nu} + \frac{1}{2\rho_0}\xi_1^2 I_1^2(\phi)n^\mu\right\}, \tag{1}$$

where $\xi_1^2 = \xi^2 = (eE_0/m\omega_0)^2$, $f_1^{\mu\nu} = n^\mu a_1^\nu - n^\nu a_1^\mu$, $a_1^\mu = (0, a_0, 0, 0)^T$ is the normalized vector potential and $n^\mu = (1, \vec{n})$. Here, $\vec{n}$ is a unit vector parallel to the laser direction of motion $\vec{n} \,||\, u_3$ and $\rho_0 \equiv (nu_0)$. Einstein notation was used (a repeated Greek index is equivalent to a summation over all dimensions and $(ab) \equiv a^\mu b_\mu$). Functions $I_1(\phi)$ and $h(\phi)$ carry the imprint of the radiation reaction. They are expressed as:

$$\begin{aligned} h(\phi) &= 1 + \frac{1}{3}\alpha\eta_0\xi^2\left[\omega_0(\phi-\phi_0) - \sin(\omega_0\phi)\cos(\omega_0\phi) + \sin(\omega_0\phi_0)\cos(\omega_0\phi_0)\right] \\ I_1(\phi) &= \cos(\omega_0\phi)h(\phi) - \cos(\omega_0\phi_0) - \frac{2}{3}\alpha\eta_0\left(\sin(\omega_0\phi) - \sin(\omega_0\phi_0)\right)\times \\ &\quad\times\left[1 + \frac{\xi^2}{3}\left(\sin^2(\omega_0\phi) + \sin(\omega_0\phi)\sin(\omega_0\phi_0) + \sin^2(\omega_0\phi_0)\right)\right], \end{aligned} \tag{2}$$

where ϕ_0 denotes the initial phase, ϕ current phase of the particle within the field of the electromagnetic wave, α is the fine structure constant, and $\eta_0 = (k_0u_0)/m$ is the wave frequency measured in the electron rest frame in units of the electron mass m. Other quantities denote elementary charge e and electromagnetic field amplitude E_0. Without radiation reaction, Eqs. (2) reduce to

$$h(\phi) = 1, \quad I_1(\phi) = \cos(\omega_0\phi) - \cos(\omega_0\phi_0). \tag{3}$$

The equations (1)-(3) are presented in natural units, as in Ref.[1]. In those units $\hbar = c = 1$, and the fine-structure constant is defined by $\alpha = e^2/\hbar c \simeq 1/137$. We now switch to the dimensionless units more common in laser-plasma interactions. Here, all the quantities are normalized to the laser frequency ω_0, such that $t \to t\omega_0$, $p \to p/mc$, $\mathscr{E} \to \mathscr{E}/mc^2$, $E \to eE/mc\omega_0$ and $B \to eB/mc\omega_0$. In these units, $\omega_0 = 1$, $\lambda_0 = 2\pi$, $c = 1$, mass is normalized to the electron mass m and charge is normalized to the elementary charge e. The particle equations of motion by component for the case without radiation reaction starting at arbitrary laser phase with an arbitrary initial momentum can be written as:

$$\begin{aligned} \gamma(\phi) &= \gamma_0 + a_0\frac{p_{0,1}}{\gamma_0 - p_{0,3}}I_1(\phi) + \frac{1}{2(\gamma_0 - p_{0,3})}a_0^2 I_1(\phi)^2 \\ p_1(\phi) &= p_{0,1} + a_0 I_1(\phi) \\ p_2(\phi) &= p_{0,2} \\ p_3(\phi) &= p_{0,3} + a_0\frac{p_{0,1}}{\gamma_0 - p_{0,3}}I_1(\phi) + \frac{1}{2(\gamma_0 - p_{0,3})}a_0^2 I_1(\phi)^2 \end{aligned} \tag{4}$$

We can compute the spatial displacement of the particle as a function of the phase ϕ. We have $x_3 = \int_0^t p_3(t)/\gamma(t)dt = \int_{\phi_0}^{\phi} p_3(\phi)/(\gamma(\phi) - p_3(\phi))d\phi$, where we used a relation between the phase and time $d\phi = dt(\gamma - p_3)/\gamma$ to perform the change of variables. The denominator is an integral of motion, such that $\gamma(\phi) - p_3(\phi) = \gamma_0 - p_{0,3}$. The spatial displacement in the laser direction of motion is then given by:

$$
\begin{aligned}
x_3 =& \frac{p_{0,3}}{\gamma_0 - p_{0,3}}(\phi - \phi_0) + \frac{a_0 p_{0,1}}{(\gamma_0 - p_{0,3})^2}\left[\sin\phi - \sin\phi_0 - (\phi - \phi_0)\cos\phi_0\right] \\
&+ \frac{a_0^2}{2(\gamma_0 - p_{0,3})^2}\left[(\phi - \phi_0)\left(\cos^2\phi_0 + \frac{1}{2}\right) - 2\cos\phi_0 \sin\phi + \frac{1}{4}\sin(2\phi) + \frac{3}{4}\sin(2\phi_0)\right]
\end{aligned}
\tag{5}
$$

The standard result for a particle starting at rest can be retrieved by taking $\gamma_0 = 1$ and $p_{0,1} = p_{0,2} = p_{0,3} = 0$. However, our particles are not created at rest. Electron-positron pairs are created by scattering an LWFA electron bunch with an intense laser at normal incidence. We can assume as a first approximation that $p_{0,1} = p_{0,3} = 0$, but $p_{0,2} \neq 0$, i.e. the particle initial velocity is perpendicular to both the laser polarization direction x_1 and to the laser propagation direction x_3.

Maximum energy and plane wave acceleration length for particles initially at 90 degrees

For a particle born at 90 degrees of incidence to the laser axis with an initial Lorentz factor γ_0, maximum attainable energy according to Eqs. (4) is given by $\mathscr{E}_{\rm max} = 2a_0^2/\gamma_0$. This is achieved when $I_1(\phi)^2$ is at the maximum. For simplicity, let us consider a particle with $\phi_0 = 0$, as an example that allows to obtain that maximum. Here, the expression for longitudinal displacement given by Eq. (5) can be simplified:

$$
x_3 = \frac{a_0^2}{2\gamma_0^2}\left(\frac{3}{2}\phi - 2\sin\phi + \frac{1}{4}\sin(2\phi)\right). \tag{6}
$$

The maximum energy $\mathscr{E}_{\rm max}$ is reached for $\phi = \pi$, after the particle has traveled a distance of $l_{\rm pwa} = 3a_0^2\pi/(4\gamma_0^2)$ or $l_{\rm pwa} = \lambda_0 \times 3a_0^2/(8\gamma_0^2)$. We define this distance as the plane wave acceleration length. If our particle would start at a different ϕ_0 in otherwise identical conditions, its maximum energy can be smaller or equal to $\mathscr{E}_{\rm max}$. For particles born at normal incidence, the particles with the lowest initial γ_0 are the ones that can attain the highest energy in the interaction with the plane wave (because $\mathscr{E}_{\rm max} \propto \gamma_0^{-1}$). However, one should note here that the higher energy gain is possible due to the slower dephasing, and the plane wave acceleration length is reversely proportional to the square of the initial energy $l_{\rm pwa} \propto \gamma_0^{-2}$. Such particles, therefore, propagate a longer distance before reaching the $\mathscr{E}_{\rm max}$.

Correction of the maximum energy due to the pulse focusing

The maximum energy and the acceleration length in the previous section are valid for a plane wave interaction. However, the lasers are not plane waves, and the laser de-focusing affects the acceleration. Particles are expected to attain an energy cutoff similar to $\mathscr{E}_{\rm max}$ in a focused laser only if $R_L \gg l_{\rm pwa}$, where $R_L = \pi W_0^2/\lambda_0$ is the Rayleigh range, W_0 and λ_0 are the laser waist and the wavelength respectively. If $R_L \simeq l_{\rm pwa}$, we require an estimate on how the laser de-focusing affects the acceleration. To this end, we perform a series of test particle simulations, where the particles are initialized with the most favourable phase for the highest energy gain within the shortest propagation distance. The test particles are born at the laser central axis, in focus, at $\phi_0 = 0$ of the wave. The 3D analytical expression for the fields of the focused laser pulse is taken from Ref.[2]. We varied the laser intensity, and the initial particle energy is chosen according to $\gamma_0 = 5 \times 10^4/a_0$ to be consistent with the lowest available energy expected in the system.

The results displayed in Fig. 1 correspond to a range of laser intensities between $a_0 = 100$ and $a_0 = 4000$ and three different waist sizes: $W_0 = 3.2$ μm, $W_0 = 5$ μm and $W_0 = 10$ μm. For every example, the maximum energy attained by the particle born in the laser focus with $\phi_0 = 0$ is divided by the maximum energy such a particle could attain in a plane wave. An interesting point to note here is that the differences between the case with and without radiation reaction are not strongly pronounced. This is consistent with the fact that a quickly acquired parallel component of the momentum reduces the χ_e and the radiation reaction stops being relevant for the particle dynamics. Figure 1 indicates that the ratio $\mathscr{E}/\mathscr{E}_{\rm max}$ is a function of $R_L/l_{\rm pwa}$ and for $R_L/l_{\rm pwa} < 1$ this can be approximated as:

$$
\frac{\mathscr{E}}{\mathscr{E}_{\rm max}} = 0.5\sqrt{\frac{R_L}{l_{\rm pwa}}} \tag{7}
$$

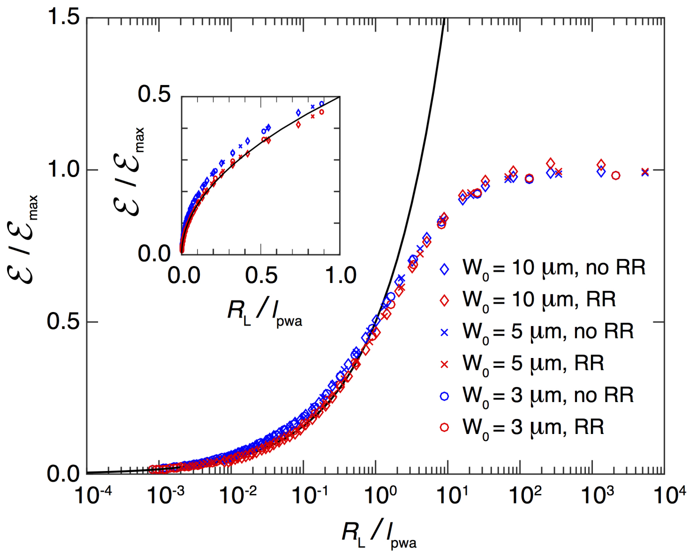

Figure 1. Ratio of the maximum attainable energy for a focused laser $\mathscr{E}$ and the maximum attainable energy for the same laser intensity in a plane wave $\mathscr{E}_{\text{max}}$ as a fuction of the ratio of the Rayleigh range R_L to the acceleration length l_{pwa}. For $0.05 < R_L/l_{\text{pwa}} < 1$, one can approximate the correction of maximum energy due to the pulse focusing using the Eq. (7).

For $R_L/l_{\text{pwa}} > 10$, we consider the condition $R_L \gg l_{\text{pwa}}$ satisfied and $\mathscr{E} \simeq \mathscr{E}_{\text{max}}$. For $l_{\text{pwa}} < R_L < 10\ l_{\text{pwa}}$, Eq. (7) is not a good estimate, due to its value being on the same order of $\mathscr{E}_{\text{max}}$. For $l_{\text{pwa}} < R_L < 10\ l_{\text{pwa}}$, the maximum energy takes values between $\mathscr{E}_{\text{max}}/2$ and $\mathscr{E}_{\text{max}}$.

Equation (7) shows the defocusing correction for the maximum energy of a particle born in the very centre of the laser field. We can assume that for an arbitrary particle born anywhere in the laser field (with the same γ_0), the effective interaction length cannot be more than twice the value for the particle born in the centre. As the effective interaction length is proportional to R_L, we should not expect to obtain particles with energies higher than

$$\frac{\mathscr{E}}{\mathscr{E}_{\text{max}}} = 0.5\sqrt{\frac{2R_L}{l_{\text{pwa}}}}. \tag{8}$$

Full-scale 3D simulations in the main manuscript are in very good agreement with the predictions for the cutoff of the particle energy spectrum given by Eq. (8).

References

1. Di Piazza, A. Exact solution of the landau-lifshitz equation in a plane wave. *Lett. Math. Phys.* **83**, 305–313 (2008). DOI 10.1007/s11005-008-0228-9.

2. Quesnel, B. & Mora, P. Theory and simulation of the interaction of ultraintense laser pulses with electrons in vacuum. *Phys. Rev. E* **58**, 3719–3732 (1998). DOI 10.1103/PhysRevE.58.3719.
